# Modulation of Immunity by Antiangiogenic Molecules in Cancer

**DOI:** 10.1155/2012/492920

**Published:** 2012-12-24

**Authors:** Magali Terme, Orianne Colussi, Elie Marcheteau, Corinne Tanchot, Eric Tartour, Julien Taieb

**Affiliations:** ^1^INSERM U970, Paris Cardiovascular Research Center (PARCC), Université Paris-Descartes, Sorbonne Paris Cité, 56 rue Leblanc, 75015 Paris, France; ^2^Service d'Immunologie Biologique, Hôpital Européen Georges Pompidou, AP-HP, 75015 Paris, France; ^3^Service d'Hépatogastro-Entérologie et d'Oncologie Digestive, Hôpital Européen Georges Pompidou, AP-HP, 75015 Paris, France

## Abstract

In the last decades a new class of therapeutic drugs have been developed that block tumor angiogenesis. These antiangiogenic molecules, which target VEGF or VEGFR, PDGFR, and c-kit, can act not only on endothelial cells but also on immune cells. Some antiangiogenic molecules inhibit the development of immunosuppressive mechanisms developed by the tumors to escape the immune system (such as regulatory T cells, myeloid-derived suppressor cells, and immunosuppressive cytokines). These immunomodulatory effects must be characterized in detail to enable a better prescription of these treatments. In this paper we will focus on the impact of anti-angiogenic drugs on immunosuppression and their potential combination with immunotherapeutic strategies. Interestingly, immune parameters or their modulation during treatment could serve as potential biomarkers of response or resistance to anti-angiogenic therapies.

## 1. Introduction

In 2000, Hanahan and Weinberg defined 6 hallmark criteria of cancer. Six points constitute these hallmarks: sustaining proliferative signaling, evading growth suppressors, resisting cell death, enabling replicative immortality, inducing angiogenesis, and activating invasion and metastasis [1]. In 2011, these criteria have been revisited and 2 emerging criteria have been added: deregulating cellular energetics and avoiding immune destruction [2]. Thus cancer cells have to bypass the surveillance of the immune system that normally restricts the development of tumors. The role of immunosurveillance in cancer has been highlighted by experimental and clinical studies. Mice deficient for lymphocytes or IFN type I signaling are more susceptible to cancer development than wild-type mice [3]. In humans, tumor incidence in immunocompromised patients (transplanted patients or HIV patients) is enhanced [4, 5]. Conversely, tumor infiltration by T or NK cells is correlated with a good prognosis in colorectal or ovarian cancer patients [6–8]. Recently, different works have shown that the immune system could play a role in the antitumor effect of conventional cancer therapies [9]. The immune system could be involved in two different ways. First, conventional cancer therapies can directly act on tumor cells and induce an immunogenic cell death. Thus, conventional cancer therapies trigger tumor cell death and release of apoptotic bodies that could be uptaken and presented by dendritic cells [9]. On the other hand, conventional cancer therapies can modulate tumor microenvironment by inhibiting immunosuppressive mechanisms induced by the tumor or by stimulating immune effector cells. In the last decade, targeted agents directed against molecular targets on cancer cells or their microenvironment have been developed. Among these targeted agents, anti-angiogenic molecules have been registered in the treatment of many types of solid tumors. They block tumor angiogenesis which is necessary to allow tumor growth and spread.

## 2. Proangiogenic Factors and Immunity

Angiogenesis is a physiological process that is involved in the formation of new blood vessels from preexisting blood vessels. It allows the supply of oxygen and nutrients and the elimination of waste. Angiogenesis represents a key event in the development of tumors. Angiogenesis is regulated by a fine balance between pro- and anti-angiogenic signals. In the absence of oxygen in the center of the tumor, hypoxia induces the expression of transcriptional factors. Among these transcriptional factors, hypoxia inducible factor (HIF) induces the expression of pro-angiogenic factors such as vascular endothelial growth factor (VEGF) and platelet-derived growth factor (PDGF). VEGF is one of the most potent sources of angiogenesis. Its expression is also controlled by different oncoproteins (such as epidermal growth factor (EGF), K-ras, PDGF, and the E6 and E7 oncoproteins of the HPV-16) [10, 11]. When proangiogenic factors are induced by hypoxia or oncoproteins, the balance between pro- and anti-angiogenic factors is deregulated resulting in an angiogenic switch that is associated with the proliferation and migration of vascular cells and the formation of new blood vessels. The structure of these new blood vessels is altered resulting in distorted and enlarged vessels, increased permeability, irregular blood flow, and microhemorrhages in the tumor. Some proangiogenic molecules such as VEGF, placental growth factor (PlGF), and hepatocyte growth factor (HGF) are able to modulate immunity [12–14]. VEGF family is composed of six different members (VEGF-A,-B,-C,-D,-E, and PlGF). VEGF-A plays a key role in the development of tumor angiogenesis and is produced by tumor cells. VEGF-A interacts with two receptors, VEGF-R1 and -R2, which are expressed on endothelial cells and also on some immune cells. VEGF-A can modulate expression of adhesion molecules on endothelial cells such as ICAM-1, -2 that are involved in leukocyte adhesion to vascular endothelium. This decrease leads to a reduced lymphocyte infiltration of the tumor [15]. VEGF-A is also involved at different levels in the induction of tumor immunosuppression. Tumor-derived VEGF-A can inhibit the activation of the transcription factor nuclear factor-*κ*B (NF-*κ*B) via VEGFR-1 signaling and thereby prevents dendritic cells (DC) maturation [12, 13, 16]. An increased VEGF plasma level is also correlated to the presence of immature DC and immature myeloid cells in the peripheral blood of cancer patients [17, 18]. Administration of exogeneous VEGF-A to tumor-free mice inhibits the differentiation of DC and promotes the accumulation of immature myeloid cells in spleen, lymph nodes, and peripheral blood [19]. VEGF-A administration also decreases splenic T cell proportion and number and suppresses their function [19]. VEGF-A-induced myeloid-derived suppressor cells (MDSC) accumulation and T-cell decrease depend on VEGFR2 signaling as assessed by administration of two VEGF_165_ variants selectively targeting VEGFR1 or VEGFR2 to tumor-free mice [20]. Proangiogenic factors could also be involved in the accumulation of regulatory T-cells in tumor-bearing mice, since induction of Treg by immature DC or immature myeloid cells such as MDSC represents one of the mechanisms of Treg differentiation in tumors. Immature DC or MDSC have the capacity to induce Treg cell proliferation in a TGF*β*- or arginase-dependent manner, respectively [21, 22]. Recently, we have shown that VEGF-A can also directly provoke Treg proliferation in a VEGFR2-dependent manner in tumor-bearing mice and metastatic colorectal cancer patients [23, 24]. VEGF-A can also contribute to tumor-associated macrophages development by inducing monocytes/macrophages recruitment to the tumor. However, other cytokines produced by the tumors such as IL-4 and IL-10 are required to induce polarization towards M2 macrophages that facilitates tumor growth, invasion, and angiogenesis [25, 26]. 

Placental growth factor (PlGF), a VEGF-R1 ligand, also impedes DC differentiation [13]. *In vitro* experiments have demonstrated that PlGF could block the capacity of human myeloid-derived DC to stimulate a Th1 response [27]. Hepatocyte growth factor (HGF) is produced by a large number of tumors (including carcinomas, soft tissue sarcoma, and hematopoietic malignancies) [28] and is implicated in tumor angiogenesis [29, 30]. c-met, the receptor of HGF, is expressed by diverse tumor cells, but can also be present at the surface of immune cells such as DC [31]. Stimulation of mouse DC with HGF inhibits their antigen-presenting function and induces a Th2 cytokine bias. These HGF-treated DC have the capacity to stimulate Treg expansion [14]. 

## 3. Anti-Angiogenic Molecules and Immunosuppression

Anti-angiogenic molecules are mainly divided into two subclasses: tyrosine kinase inhibitors that target receptors of proangiogenic molecules and block their signaling pathway and the monoclonal antibodies that directly target circulating proangiogenic factors or their receptors. Among tyrosine kinase inhibitors, sunitinib and sorafenib have been approved for the treatment of different tumor types including metastatic renal carcinoma and hepatocellular carcinoma. Sunitinib targets VEGFR1-3, PDGFR, c-kit, and Flt3 [32], whereas sorafenib blocks VEGFR1-3, PDGFR, c-kit, Raf-kinases [33]. Bevacizumab, a monoclonal antibody that specifically targets VEGF-A, is commonly used to treat different tumor types including metastatic colorectal cancer patients [34, 35]. These anti-angiogenic molecules have been described to act both on immunosuppression and immune responses. Bevacizumab has, for example, been shown to slightly enhance DC proportion and function (allostimulatory capacity and cytokine production) in solid cancer patients [18]. This result was in accordance with mouse studies where anti-VEGF antibody enhances number and functions of DC [36, 37]. Sunitinib-treated mice revealed a modification of their tumor microenvironment characterized by a reduced expression of immunosuppressive cytokines (IL-10, TGF*β*) and inhibitory molecules (PD-1, CTLA-4) [38].

Beyond these results the action of anti-angiogenic therapies on tumor-induced immunosuppression has been mainly studied through their impact on Treg and MDSC.

### 3.1. Regulatory T Cells

 Sunitinib treatment results in a decrease of Treg percentage and number in spleens and tumors in different mouse tumor models and in the peripheral blood and tumors of mRCC patients [38, 39]. Same observations have been reported after sorafenib treatment in mouse tumor models and in the peripheral blood of mRCC patients and hepatocellular carcinoma patients with liver cirrhosis [40–44]. In mRCC patients treated with sunitinib who had an elevated percentage of Treg at baseline, a progressive decrease in Treg was observed during treatment. This decrease began after the first cycle of treatment and became statistically significant after the second cycle [45]. Preliminary data on bevacizumab, an anti-VEGF-A antibody, showed that in three out of four metastatic colorectal cancer patients, anti-VEGF-A treatment resulted in a Treg decrease in peripheral blood [46]. In another study performed on mRCC patients bevacizumab treatment associated with low-dose IL-2 enhanced Treg proportion in peripheral blood [47]. The discrepancies of these results could be explained by the ability of low doses of IL-2 to induce Treg proliferation [48]. We recently demonstrated that targeting VEGF-A is sufficient to modulate Treg proportion and number in cancer since anti-VEGF-A antibody (in mice) or bevacizumab (in humans) inhibits Treg increase in a mouse model of colorectal cancer and in metastatic colorectal cancer patients, respectively [23]. Different mechanisms involved in the modulation of Treg by anti-angiogenic drugs targeting VEGF-A/VEGFR are proposed. Tumor-derived Treg expansion is usually attributable to four different mechanisms: preferential migration towards tumor via hypoxia which induced a special set of chemokines preferentially recruiting Treg [49], conversion of conventional T cells into Treg, proliferation of preexisting Treg, and preferential survival of Treg in oxidative stress-mediated cell death compared to conventional T cells [50–52]. Correlative studies have suggested that sunitinib-induced Treg decrease could be due to a reduction of myeloid-derived suppressor cells (MDSC) [50]. Sunitinib could also block the conversion of conventional CD4^+^Foxp3^−^ T cells into CD4^+^Foxp3^+^ Treg [53]. We have shown that targeting VEGF-A/VEGFR could also inhibit the VEGF-A-induced proliferation of Treg in colorectal cancer models and patients [23]. 

### 3.2. Myeloid-Derived Suppressor Cells

Sunitinib treatment has been observed to decrease MDSC proportion in spleens and tumors of different mouse tumor models [38, 54]. Sunitinib seems to modulate MDSC by two different ways. This tyrosine kinase inhibitor inhibits the proliferation of the monocytic subset of MDSC (Gr1^lo^) and induces the apoptosis of the granulocytic subset (Gr1^hi^) [55]. In mRCC patients, after the first cycle of sunitinib treatment the proportion of all MDSC subsets (immature lin^−^, monocytic CD33^+^CD14^+^DR^−^, and granulocytic CD33^+^CD15^+^DR^−^ MDSC) was reduced in the peripheral blood [50]. Sorafenib also reduces MDSC proportion in a mouse model of liver carcinoma and in mammary tumors as assessed, respectively, by flow cytometry and immunohistochemical stainings [41, 56]. In a mouse model of renal cancer, anti-VEGF antibody treatment decreases the number of CD11b^+^VEGFR1^+^myeloid cells that are able to suppress T-cell responses [57]. In cancer patients, the impact of bevacizumab is controversial. A study performed on solid cancer patients describes a decrease of immature myeloid cells (CD45^+^lin^−^HLA-DR^−^) after one cycle of bevacizumab treatment [18]. However, in mRCC patients, bevacizumab alone had no effect on MDSC proportion. An increase of MDSC proportion was even observed after addition of IL-2 to bevacizumab regimen [58]. It is of note that the phenotypic markers used in these two studies were different. In the study performed in solid cancer patients, immature MDSC were characterized using CD45^+^lin^−^HLA-DR^−^ staining, whereas in mRCC patients granulocytic MDSC were analyzed using CD66b marker. These results may suggest that bevacizumab could modulate not all MDSC subsets but only immature MDSC [18, 58]. The impact of IL-2 on MDSC proportion could also not be excluded [59]. In a mouse model of melanoma, one study showed a critical role for tumor-expressed iNOS in the recruitment of MDSC via the modulation of VEGF secretion [60]. As other cytokines or growth factors (IL-6, IL-10, EGF, and HGF), VEGF could activate the transcription factor STAT3 which plays a key role in cancer. STAT3 promotes survival and proliferation of tumor cells, angiogenesis, and metastasis but also the development of immunosuppressive pathways [61]. The capacity of sunitinib to block STAT3 phosphorylation has been recently described. STAT3 inhibition could at least in part explain the effect of sunitinib on MDSC reduction [54]. 

## 4. Immune Recovery after Anti-Angiogenic Therapies

Although anti-angiogenic molecules can inhibit the development of immunosuppressive mechanism (Treg, MDSC, immunosuppressive cytokines, and inhibitory molecules), the immunostimulatory capacity of these molecules is a crucial point to induce an efficient antitumor immune response. 

### 4.1. Tumor Infiltration by Immune Cells

Sunitinib treatment has the capacity to enhance the CD4^+^ T and CD8^+^ T cell intratumoral infiltration in mice [38], and VEGF/VEGFR2 blockade has been shown to improve the infiltration of adoptively transferred T cells into the tumor [62]. Anti-VEGFR2 treatment could also enhance migration of tumor-specific T cells induced by a vaccination strategy to the tumor [63]. These observations could be explained by the normalization of tumor vasculature observed during antiangiogenic treatments and prevention of ICAM-1 and V-CAM-1 downregulation on tumor endothelial cells [64].

### 4.2. T-Cell Modulation

Sunitinib and sorafenib do not have the same impact on T-cell activation. Sunitinib treatment enhances the functional capacity of tumor infiltrating T cells by stimulating IFN*γ* production and cytolytic activity against the tumor [38] and improves Th1 response after stimulation of PBMC by anti-CD3/CD28 antibodies in mRCC patients [39, 50]. This may be due to the MDSC and Treg proportion decrease in the peripheral blood of these patients. Concerning sorafenib, it seems to suppress T-cell activation and proliferation. In PBMC obtained from healthy volunteers, sorafenib inhibits CD25 and CD69 expression on T cells and IL-2 production after PHA stimulation and induces T-cell apoptosis *in vitro* [65]. Sorafenib treatment can reduce Lck phosphorylation in the context of T cell stimulation which could explain the inhibition of T-cell activity [65]. Sorafenib also targets raf-1 that is involved in T-cell activation induced by TCR engagement [66]. Raf-1 blockade by sorafenib might also result in T cell inhibition. Sorafenib could also inhibit DC maturation in response to TLR stimulation and their functional capacity especially cytokine secretion [67]. Thus, sunitinib and sorafenib seem to modulate T cell function differently. The spectrum of activity of these two molecules is different and could account for these discrepancies. However, studies showing inhibition of T-cell activity by sorafenib have been performed *in vitro*. In two studies performed on mRCC patients or liver cirrhosis patients with advanced hepatocellular carcinoma treated with sorafenib, no impact on Th1 response has been observed [42, 44]. In metastatic colorectal cancer patients, bevacizumab treatment enhances absolute number of CD4, CD8, and CD3 lymphocytes [68] and IL-2 and IFNg production after restimulation of PBMC by anti-CD3 antibody [69].

### 4.3. NK Cells

NK cells are involved in the control of tumor development and could be responsible for the development of an efficient antitumor immune response. In an *in vitro* study, sorafenib decreases NK cell reactivity against tumor cells by downregulating PI3Kinase and Erk1/2 phosphorylation [70]. However, high concentrations of sorafenib have been used in this study that do not seem to correspond to physiological doses. Sunitinib modulates neither NK cell percentage in the peripheral blood of mRCC patients nor their functions *in vitro* [70, 71]. However, expression of ligands of NK cell activating receptors on tumor cells could be regulated by tyrosine kinase inhibitors. Thus, sunitinib and sorafenib induce NKG2D ligand expression on nasopharyngeal carcinoma and hepatocellular carcinoma cell lines which confers an enhanced sensitivity to NK cell lysis [72, 73]. Huang et al. demonstrated that sorafenib decreases expression of ADAM9, a metalloproteinase that is involved in the shedding of major histocompatibility complex class I-related chain A (MICA), a ligand of NKG2D [72]. It induces a decrease of soluble MICA production and an increase in membrane-bound MICA expression. 

## 5. Synergy between Anti-Angiogenic Therapies and Cancer Vaccines

Anti-angiogenic molecules modulate immunosuppression induced by the tumor, enhance Th1 response after mitogenic restimulation, and increase T-cell infiltration into the tumors but they do not seem to be able to restore a spontaneous specific T-cell response to tumor antigens. Thus, in a mouse model of colorectal cancer expressing the carcinoembryonic antigen (CEA), sunitinib treatment does not induce a CEA-specific T-cell response [74]. However, strategies inhibiting tumor-induced immunosuppressive mechanisms create permissive conditions to induce an efficient anti-tumor immune response after vaccination. Different approaches are commonly used to eliminate Treg in tumor-bearing mice. Among these approaches, metronomic doses of cyclophosphamide or antagonists of CCR4 boost the development of an efficient anti-tumor immune response induced by the vaccination [75, 76]. But strategies commonly used to deplete or block Treg functions have major drawbacks: depletion of all Treg elimination of activated effector T cells. Anti-angiogenic molecules appear to have several advantages: (1) they only restore Treg proportion to a physiological level avoiding autoimmune mediated side effects; (2) they do not deplete activated T cells; (3) they inhibit other immunosuppressive pathways such as MDSC; (4) they have potential anti-tumor effects on their own. Different vaccination strategies have been tested in combination with anti-angiogenic therapies in mouse tumor models. Adenoviral vectors expressing soluble VEGFR-1 and -2 associated with a GM-CSF-secreting tumor cell immunotherapy enhance anti-tumor immune response in mouse models of colorectal cancer and melanoma [77]. Recombinant viral vectors expressing tumor antigen or costimulation molecules have also been associated with anti-angiogenic molecules. Sunitinib enhanced anti-tumor effects of an adenoviral vector expressing IL-12 coadministrated with 4.1BB ligand, a costimulatory molecule [38]. In a model of hepatitis virus-induced hepatocellular carcinoma in eastern woodchuck, administration of two adenoviral vectors, one encoding GM-CSF and IL-12 and the other expressing factors with anti-angiogenic properties (endostatin and pigment epithelium-derived factor), decreased tumor volume. This combination results in an enhancement of NK cell infiltration and a decrease of immunoregulatory molecules CTLA-4 and PD-1 as assessed by RT-PCR studies [78]. Association of sunitinib with poxvirus vaccination encoding for B7-1, ICAM-1, LFA-3 and the CEA antigen promoted the enhancement of CEA-specific CD8^+^ T cells in the tumor. Peptide-pulsed DC have been combined to anti-VEGF antibody or sunitinib treatment resulting in an induction of specific CD8^+^ T cells in tumor draining lymph nodes or tumors [36, 79]. Administration of sunitinib before vaccination induced a superior anti-tumor efficacy than administration after vaccination or concurrently [74]. This synergy could be explained by the modulation of immunosuppression induced by the tumor which results in a better induction of antigen-specific CD8^+^ T cells after vaccination. Anti-angiogenic molecules could also transiently normalize tumor vascularization [80]. This vascular normalization could help CD8^+^ T cell influx in the tumor after vaccination [63]. Vascular normalization could also be accompanied by a decrease of hypoxia. Since hypoxia could be involved in the development of immunosuppressive mechanisms, the suppression of hypoxia could also be a mechanism of modulation of tumor-induced immunosuppression [81]. In a mouse mammary tumor model, anti-VEGFR2 antibody enhances tumor perfusion and thereby decreases hypoxia, leading to a more efficient CD8^+^ T cell infiltration and modulation of immunosuppression. These 2 phenomenon (modulation of immunosuppression and vascular normalization) seem to be linked [81]. However, we could hypothesize that the synergys between anti-angiogenic molecules and vaccination could not be only due to revascularization and a better influx of CD8^+^ T cells to the tumor since association of anti-angiogenic with vaccine only synergize when anti-angiogenic molecules are administered before vaccination and not at the same time [74]. 

## 6. Biomarkers for Response to Anti-Angiogenic Therapies

Anti-angiogenic drugs can cause side effects (especially cardiovascular, renal, or skin adverse effects) or amplify adverse effects of chemotherapy. Thus, determining predictive markers of efficacy and tolerability would be helpful to give the right treatment to the right patient and avoid undue health costs and toxicities. The anti-angiogenic action of currently registered molecules could be transient, and secondary resistance to anti-angiogenic molecules can occur during the treatment. Detection of the occurrence of resistance could assist with prompt modification of therapeutic protocols, thereby preventing the continued administration of a treatment that no longer benefits the patient.

There are currently no validated biomarkers used in daily practice to predict efficacy, occurrence of secondary resistance, or even tolerability in patients treated with AA therapies. Many potential biomarkers are under investigation, some are measured before treatment at baseline, the others during the treatment, including second generation tests measuring plasma VEGF levels, CEC, PET scan imaging, or specific immunohistochemical stainings [82, 83]. 

Other types of predictive biomarkers to be explored may be immune cells or cytokines immunomonitoring as anti-angiogenic molecules modulate immune parameters in peripheral blood. These immune parameters could be routinely analyzed on dedicated platforms. We have recently shown that after 3 cycles of treatment with sunitinib we observed a decrease of Treg in peripheral blood in 28 mRCC patients. Patients who exhibit a 10% decrease of Treg after the third cycle of sunitinib treatment showed a better overall survival than the other patients [45]. However, a correlation between Treg decrease and clinical response was not observed when other clinical criteria have been used, such as RECIST criteria or progression-free survival. Other immune parameters could help to detect refractoriness to anti-angiogenic drugs. Resistance to anti-VEGF treatment coincided with an increase of tumor infiltration by MDSC in different mouse tumor models [84]. IL-8 has also been implicated as an escape mechanism to sunitinib treatment. An increase of plasmatic level of IL-8 has been detected in different preclinical tumor models resistant to sunitinib. Administration of neutralizing anti-IL-8 antibody resulted in the restoration of tumor sensitivity to anti-angiogenics [85]. Intratumoral IL-8 level was also enhanced in mRCC patients that did not respond to sunitinib [85]. Finally, an increase in HGF and bFGF plasma levels has been observed prior to progression in a cohort of 43 metastatic colorectal cancer patients treated by bevacizumab [86]. Thus immune biological parameters may be interesting to predict anti-angiogenic efficacy. However, these potential biomarkers have to be validated in large prospective clinical trials. Standardized techniques are also necessary for their routine use. 

## 7. Conclusions 

Anti-angiogenic molecules have immunomodulatory effects in mouse tumor models and cancer patients. However, all anti-angiogenic drugs did not have the same impact on the immune system, probably depending on their targets. Precise mechanisms involved in the influence of anti-angiogenic on the immune system are not fully understood. In the future, we need to consider the immunomodulatory effects of these targeted therapies in anticancer strategies to better prescribe these drugs. Some of the anti-angiogenic and especially sunitinib could be successfully associated with immunotherapeutic strategies. Furthermore, the immunomodulatory properties of these molecules could be used in other diseases than cancer, as autoimmune or graft-versus-host disease. Finally, defining immune biomarkers to predict anti-angiogenic response could also be useful to better select patients and avoid unnecessary adverse events and undue cost. 
